# Bridging the Gap: A Systematic Review of Digital Consent Tools to Enhance Participation and Procedures in Low-Resource Settings

**DOI:** 10.7759/cureus.94280

**Published:** 2025-10-10

**Authors:** Kiranjot Kaur, Raja Waqas, Harleen Kaur, Mashal Mumtaz, Nimrah Majeed, Shabeh e Roshan Ali, Kinza Ali, Shenouda Shehata Abdelmesih, Lubna Rehman

**Affiliations:** 1 General Practice, US Navy, United States Military, Great Lakes, USA; 2 Clinical Research, Arizona State University, Tempe, USA; 3 Internal Medicine, Shri B M Patil Medical College, Bijapur, IND; 4 Regulatory Sciences and Health Safety, Arizona State University, Arizona, USA; 5 Internal Medicine, Shri Guru Ram Rai Institute of Medical and Health Sciences, Dehradun, IND; 6 Internal Medicine, University College of Medicine and Dentistry, University of Lahore, Lahore, PAK; 7 General Medicine, Abbasi Shaheed Hospital, Karachi, PAK; 8 General Medicine, Cavan and Monaghan Hospital, Cavan, IRL; 9 Medicine and Surgery, Liaquat University of Medical and Health Sciences, Jamshoro, PAK; 10 Internal Medicine, Cavan General Hospital, Cavan, IRL; 11 Medicine and Surgery, Civil Hospital Karachi, Karachi, PAK; 12 Nephrology, Liaquat National Hospital, Karachi, PAK; 13 Orthopedics and Traumatology, Khoula Hospital, Muscat, OMN; 14 General Medicine, Dow University of Health Sciences, Civil Hospital Karachi, Karachi, PAK

**Keywords:** digital consent, e-consent, informed consent, low-resource settings, participation

## Abstract

Informed consent is central to ethical clinical research, yet traditional paper-based processes are often lengthy, complex, and literacy-dependent, disproportionately disadvantaging participants in low-resource settings. This review assessed the role of digital consent tools (e-consent) in enhancing comprehension, satisfaction, and documentation quality in low-resource settings, while also examining barriers, facilitators, and scalability. Given the small number of studies identified, findings should be interpreted as preliminary. Following Preferred Reporting Items for Systematic reviews and Meta-Analyses (PRISMA) guidelines, PubMed, Embase, Scopus, and Cochrane were searched up to August 2025 for randomized and non-randomized trials, observational studies, pilot projects, and systematic or scoping reviews comparing e-consent with traditional methods. Risk of bias was assessed using ROBINS-I for non-randomized studies, AMSTAR-2 for systematic reviews, and the JBI tool for scoping reviews, with findings synthesized narratively. Ultimately, only six studies met the inclusion criteria, underscoring the limited evidence base in this area. Multimedia, offline tablet-based, web-based, and AI-assisted e-consent platforms consistently improved comprehension and satisfaction, while documentation errors decreased markedly in low-resource contexts. Effects on enrollment were mixed, with outcomes influenced by trial protocols and platform usability. Reported barriers included low digital literacy, connectivity challenges, and heterogeneity of tools, whereas facilitators included multilingual adaptability, offline compatibility, and integration into clinical workflows. Overall, e-consent demonstrates strong potential to reduce inequities in the consent process, provided systems are adapted to local cultural, literacy, and infrastructural contexts and aligned with international regulatory standards.

## Introduction and background

Informed consent is the foundation of ethical clinical practice and research. It represents not only a legal requirement but also an ethical safeguard to ensure autonomy, transparency, and trust between participants and investigators. The classical consent process emphasizes disclosure of information, comprehension, voluntariness, and documentation [1]. However, despite its central role, traditional paper-based consent forms often fail to achieve true understanding, with studies showing that many participants recall less than half of critical trial information after signing consent documents. Traditional consent approaches have been criticized for their complexity, excessive length, and reliance on literacy, which disproportionately disadvantage populations in low-resource and diverse cultural settings. Long textual forms may overwhelm patients, while logistical challenges in documentation and archiving can compromise both patient rights and institutional accountability [2]. These barriers underscore the need for more accessible, adaptive approaches to informed consent. The advent of electronic informed consent (e-consent) has transformed this process. E-consent integrates multimedia tools, such as interactive graphics, audio narration, video explanations, and digital signatures, to improve participant engagement and comprehension.

Studies in clinical research have shown that e-consent enhances understanding, improves satisfaction, and reduces missing or erroneous documentation compared to traditional methods [3]. Importantly, e-consent also facilitates remote participation, an aspect highlighted during the COVID-19 pandemic, where digital alternatives became essential for maintaining research continuity. Recognizing these benefits, international regulatory bodies have developed guidelines for the use of e-consent. The U.S. Food and Drug Administration (FDA) and the Office for Human Research Protections (OHRP) published joint recommendations outlining requirements for the validity, authentication, and secure storage of digital consent records, emphasizing participant comprehension as the cornerstone of ethical acceptability [4]. Similarly, the European Medicines Agency (EMA) has acknowledged the role of e-consent in harmonizing clinical trial procedures across diverse health systems [5]. Methods of e-consent vary widely: from simple web-based consent forms and mobile tablet applications to sophisticated interactive modules integrated with audiovisual aids and AI-driven explanations. These approaches not only standardize the consent process but also provide audit trails and multilingual adaptability, enabling researchers to tailor communication to participant needs.

Despite these advancements, applying e-consent in low-resource settings presents unique challenges. Limited internet connectivity, low digital literacy, and infrastructural gaps can impede implementation. Yet, such contexts may benefit most from these tools, given the prevalence of literacy barriers, understaffing, and weak documentation systems. By leveraging offline-compatible systems, multimedia translations, and culturally appropriate adaptations, digital consent tools may bridge the participation gap and reduce procedural inequities in these regions. To systematically evaluate the evidence on digital consent tools in low-resource healthcare and research settings, with a focus on their impact on participation, comprehension, satisfaction, and documentation quality, while identifying barriers, facilitators, and pathways for scalable implementation.

## Review

Materials and methods

Search Strategy

A systematic literature search was conducted following the PRISMA (Preferred Reporting Items for Systematic Reviews and Meta-Analyses) guidelines [6]. We searched PubMed, Embase, Scopus, and the Cochrane Library for studies published up to August 2025 using combinations of the terms digital consent, e-consent, electronic informed consent, low-resource, developing countries, and clinical trials. References of included studies and relevant reviews were also hand-searched to identify additional eligible articles.

Eligibility Criteria

Eligibility was defined using the PICO framework [7].

Population (P): Underserved or low-resource populations, with a focus on rural and suburban settings. Only adult participants aged 18 years and older were considered.

Intervention (I): Digital or electronic informed consent tools, including multimedia, web-based, mobile, offline-compatible, or AI-assisted systems.

Comparator (C): Traditional paper-based informed consent methods.

Outcomes (O): Participation rates, comprehension, satisfaction, retention, feasibility, and documentation quality.

Studies were included if they evaluated digital consent tools in healthcare or clinical research and were designed as randomized controlled trials, non-randomized interventional studies, observational studies, pilot projects, or systematic reviews. Case reports, editorials, and conference abstracts were excluded.

Study Selection

Two reviewers independently screened all titles and abstracts for relevance. Full texts of potentially eligible studies were retrieved and reviewed in detail. Disagreements were resolved by discussion and consensus.

Data Extraction

A standardized data extraction form was developed to record study characteristics, participant demographics, intervention type, comparator, outcomes assessed, and barriers or facilitators reported. Extracted data were cross-checked by two reviewers for accuracy.

Risk of Bias Assessment

Risk of bias was assessed using validated tools tailored to the study design. Observational and non-randomized studies were evaluated using ROBINS-I [8], systematic reviews using AMSTAR-2 [9], and scoping reviews using the Joanna Briggs Institute (JBI) Critical Appraisal Tool [10]. Risk ratings were independently assigned, with disagreements resolved through consensus.

Data Synthesis

Given the heterogeneity of study designs, populations, interventions, and outcomes, a narrative synthesis was undertaken instead of a meta-analysis. Studies were organized by design (randomized trials, observational studies, pilot projects, and systematic or scoping reviews) and compared across key outcomes, including participation rates, comprehension, satisfaction, documentation quality, and feasibility. Barriers and facilitators were analyzed thematically, with particular attention to digital literacy, infrastructural limitations, and cultural factors relevant to underserved and low-resource settings. Findings were triangulated across primary studies and systematic reviews to identify consistent patterns, highlight contextual differences between high-income and low-resource settings, and assess implications for scalability and ethical adoption of digital consent tools.

Results

Study Selection Process

**Figure 1:**
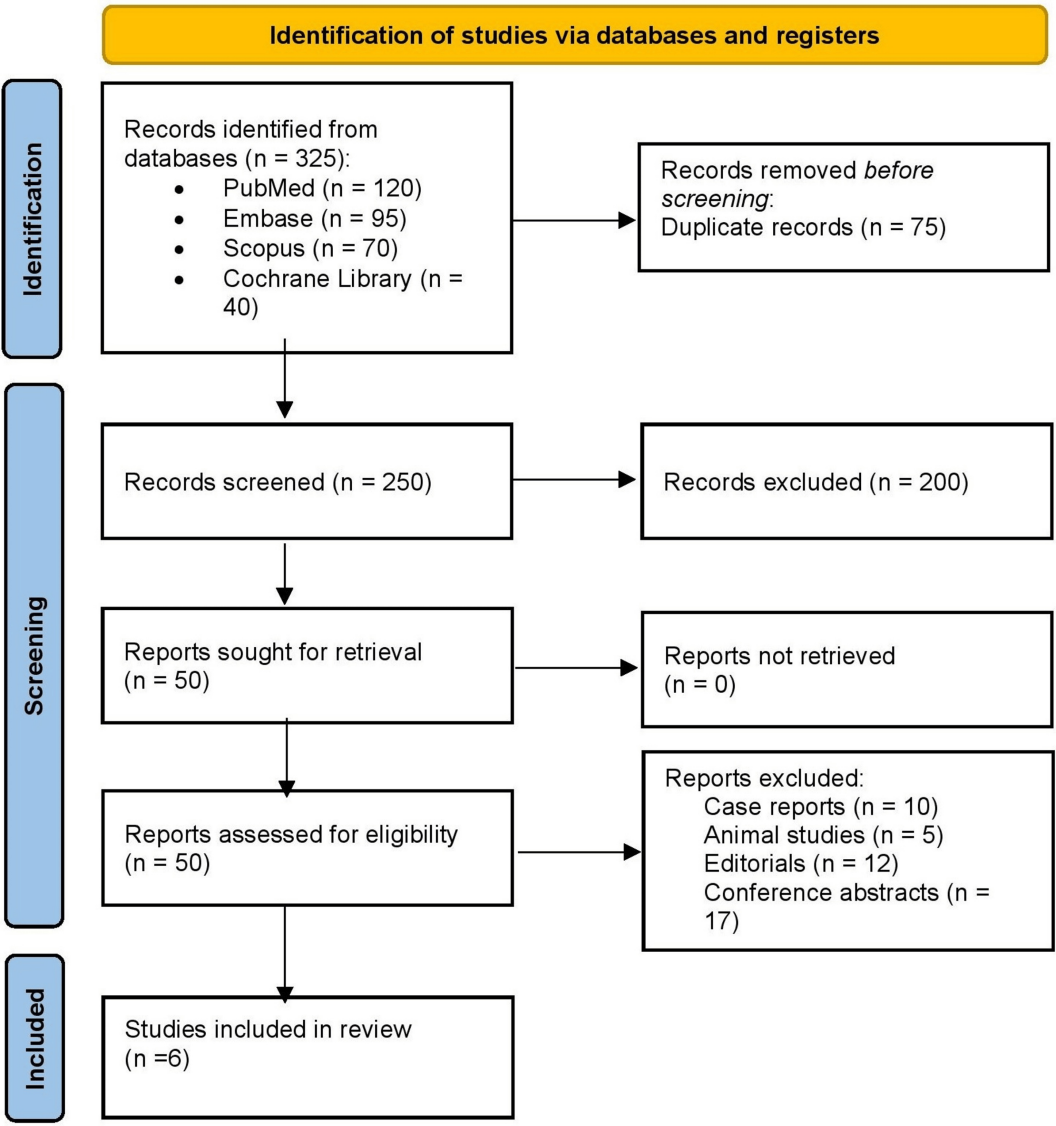
PRISMA 2020 flow chart PRISMA: Preferred Reporting Items for Systematic reviews and Meta-Analyses

Figure 1 shows that a total of 325 records were identified through database searching: PubMed (n = 120), Embase (n = 95), Scopus (n = 70), and Cochrane Library (n = 40). After removing 75 duplicates, 250 records remained for title and abstract screening. Of these, 200 records were excluded as irrelevant to the research question. The remaining 50 full-text articles were retrieved and assessed for eligibility. During full-text review, 44 articles were excluded for the following reasons: case reports (n = 10), animal studies (n = 5), editorials (n = 12), and conference abstracts (n = 17). Ultimately, six studies met the inclusion criteria and were included in the final synthesis.

Characteristics of the Selected Studies

Table 1 presents the characteristics of the six studies included in this review, spanning diverse geographical and clinical contexts. Ngoliwa et al. (2025) conducted an observational pilot study in Malawi, demonstrating that tablet-based offline e-consent tools eliminated documentation errors compared with paper forms [11]. Mazzocchi et al. (2023) synthesized data from high-income multicenter RCTs, showing improved comprehension but variable effects on enrollment [12]. In Nigeria, Afolabi et al. (2014) piloted a multimedia consent tool in low-literacy rural populations, which significantly enhanced understanding and satisfaction [13]. Goldschmidt et al. (2025) explored AI-driven and web-based tools across European hospital sites, highlighting improvements in workflow and comprehension but limited Low- and Middle-Income Country (LMIC)-specific data [14]. Gesualdo et al. (2021) systematically reviewed multimedia approaches, reporting consistent gains in comprehension and satisfaction across heterogeneous trial populations [15]. Similarly, Cohen et al. (2023) assessed participants in clinical trials, concluding that digital platforms improved comprehension and documentation quality, though feasibility varied across sites [16]. Collectively, these findings underscore both the promise and contextual challenges of e-consent in enhancing informed consent processes.

**Table 1:** Characteristics of the selected studies RCT = Randomized Controlled Trial; LMIC = Low- and Middle-Income Country; AI = Artificial Intelligence; e-Consent = Electronic Consent; ODK = Open Data Kit

Author / Year	Setting	Study Design	Population (P)	Intervention (I)	Comparator (C)	Outcomes (O)	Barriers / Facilitators	Key Findings
Ngoliwa et al., 2025 [11]	Malawi / Tertiary hospital	Observational pilot	109 adult patients	E-consent via Open Data Kit tablets	Paper-based consent	Participation rate, documentation errors, usability	Limited digital literacy; facilitator: offline-friendly tool	100% uptake with e-consent; eliminated documentation errors vs 43% error rate in paper forms
Mazzocchi et al., 2023 [12]	Multicenter / High-income RCTs	Systematic review	8864 Participants	Electronic informed consent systems	Standard paper consent	Enrollment rates, comprehension, and retention	Variability in trial protocols; facilitator: user-friendly digital platforms	e-Consent improved comprehension and recall; mixed effects on enrollment
Afolabi et al., 2014 [13]	Nigeria / Rural LMIC	Experimental trial tool	42 Low-literacy rural participants	Multimedia consent tool (audio-visual)	Standard consent	Understanding, satisfaction	Barrier: literacy, language; facilitator: visual/audio translation	Improved understanding in low-literacy groups; higher satisfaction compared to standard
Goldschmidt et al., 2025 [14]	Europe / Mixed hospital sites	Scoping review	Clinical staff & patients in 27 studies	AI/web-based consent tools	Standard consent	Comprehension, satisfaction, workflow impact	Barrier: infrastructure; facilitator: mobile/web integration	Digital tools supported workflow, improved comprehension; limited LMIC-specific evidence
Gesualdo et al., 2021 [15]	Global / Systematic review	Systematic review	Mixed clinical trial populations of 73 studies	Multimedia consent approaches	Traditional text consent	Participation, satisfaction, comprehension	Barrier: heterogeneity of tools; facilitator: multimedia interactivity	Multimedia tools consistently improved satisfaction and comprehension
Cohen et al., 2023 [16]	International / Clinical trials	Systematic review	13,281 participants	e-Consent digital platforms	Paper consent	Effectiveness, comprehension, and feasibility	Barrier: diverse tech readiness; facilitator: centralized platforms	Digital e-consent improved comprehension and documentation; feasibility varied across sites

Risk of Bias Assessment

Table 2 shows that most studies had a moderate risk of bias. Ngoliwa et al. (2025) and Afolabi et al. (2014) were limited by small, non-randomized designs [11,13]. The systematic reviews by Mazzocchi et al. (2023), Gesualdo et al. (2021), and Cohen et al. (2023) were rated low to moderate due to strong methods but high heterogeneity [12,15,16]. Goldschmidt et al. (2025) was also rated moderate, reflecting its descriptive approach [14]. Overall, evidence was consistent despite methodological variability.

**Table 2:** Risk-of-bias assessment ROBINS-I = Risk Of Bias In Non-randomized Studies of Interventions; AMSTAR-2 = A MeaSurement Tool to Assess Systematic Reviews 2; JBI = Joanna Briggs Institute; RoB = Risk of Bias

Study	Study Design	Risk of Bias Tool	Risk of Bias Rating	Justification
Ngoliwa et al., 2025 [11]	Observational pilot	ROBINS-I	Moderate	Non-randomized design; limited sample size; strong outcome clarity but pilot data only.
Mazzocchi et al., 2023 [12]	Systematic review	AMSTAR-2	Low–Moderate	Predefined protocol; broad search; high heterogeneity in included trials limited synthesis.
Afolabi et al., 2014 [13]	Experimental study	ROBINS-I	Moderate	Targeted low-literacy setting; good design but small sample and no blinding.
Goldschmidt et al., 2025 [14]	Scoping review	JBI Critical Appraisal Tool	Moderate	Narrative synthesis; lacks quantitative rigor; descriptive focus only.
Gesualdo et al., 2021 [15]	Systematic review	AMSTAR-2	Low–Moderate	Comprehensive search strategy; outcomes heterogeneous; some risk of reporting bias.
Cohen et al., 2023 [16]	Systematic review	AMSTAR-2	Low–Moderate	Strong methodology; diverse study designs reduced comparability of pooled findings.

Discussion

This systematic review highlights the growing role of electronic informed consent (e-consent) in modern healthcare and clinical research, particularly in addressing barriers inherent in traditional paper-based systems. Conventional consent processes often overwhelm participants with complex text, depend heavily on literacy, and suffer from documentation errors or omissions. These challenges are amplified in low-resource settings, where literacy levels, limited infrastructure, and weak archiving systems hinder both participant comprehension and institutional accountability. Evidence from the included studies reinforces these advantages. In Malawi, Ngoliwa et al. demonstrated that an offline tablet-based e-consent system achieved full uptake and eliminated documentation errors, showing its feasibility in a low-resource environment [11]. Afolabi et al. found that multimedia consent tools using audio-visual aids significantly improved comprehension and satisfaction among rural, low-literacy populations in Nigeria [13]. At a broader level, Mazzocchi et al. and Cohen et al. both confirmed through systematic reviews that e-consent improves comprehension and documentation across diverse clinical trials, although effects on enrollment rates were variable [12,16].

Gesualdo et al. further supported the role of multimedia approaches in enhancing satisfaction and understanding across heterogeneous trial populations [15]. Meanwhile, Goldschmidt et al. highlighted the promise of AI- and web-based systems in Europe, particularly in improving workflow and comprehension, though evidence from LMICs remains limited [14]. Collectively, these studies suggest that while effectiveness is consistent, context-specific challenges shape feasibility and scalability. E-consent introduces multimedia and interactive technologies that transform the consent process from a static legal requirement into a dynamic, participant-centered exchange. Methods vary in sophistication and delivery. Web-based platforms allow remote access to consent materials and can be integrated with centralized trial databases. Tablet-based and offline-compatible systems are particularly relevant in low-resource settings, where internet connectivity is unreliable; the study in Malawi demonstrated how such tools can achieve near-universal uptake and eliminate documentation errors [17]. Multimedia tools, such as video explanations, audio narration, and pictorial aids, directly address barriers posed by low literacy, as shown in Nigeria, where comprehension and satisfaction improved in rural participants. AI-driven platforms are emerging innovations, providing adaptive explanations based on real-time participant feedback and literacy levels [18]. These systems offer potential for personalization, though evidence of their effectiveness in LMICs remains limited.

Beyond comprehension, e-consent has been shown to improve retention of information, participant satisfaction, and auditability of the consent process. Standardization through digital records also strengthens regulatory compliance, providing secure, time-stamped documentation trails that reduce legal and ethical risks. During the COVID-19 pandemic, the scalability of remote e-consent demonstrated its value in maintaining research continuity while minimizing face-to-face contact. Importantly, international bodies such as the FDA, OHRP, and EMA have recognized e-consent as a valid and ethically acceptable alternative, provided that core principles of autonomy, comprehension, and voluntariness are upheld. Nevertheless, implementation is not without challenges. Digital literacy varies widely, and in underserved populations, unfamiliarity with technology may paradoxically reduce comprehension if interventions are not designed with cultural and contextual sensitivity. Infrastructure gaps, such as unreliable electricity and internet access, remain major obstacles in rural regions. Ethical concerns also persist regarding data security, confidentiality, and the risk of coercion if digital tools are presented in a non-neutral or overly persuasive format. Moreover, heterogeneity in design and delivery across studies complicates direct comparisons and hinders meta-analytic synthesis.

In summary, e-consent represents a significant advancement over paper-based methods, with multimedia, offline, web-based, and AI-assisted approaches each offering distinct advantages. While evidence supports its ability to improve comprehension, satisfaction, and documentation, widespread adoption requires careful adaptation to local contexts, regulatory alignment, and safeguarding of privacy and autonomy. Limitations of this review include the small number of eligible studies and the heterogeneity of interventions, which precluded quantitative synthesis. Future research should focus on scalable, context-specific models tailored to underserved populations.

## Conclusions

This review highlights that electronic informed consent (e-consent) provides advantages over traditional paper-based methods by improving participant understanding, satisfaction, and documentation quality. Multimedia features, offline access, web platforms, and AI-assisted systems are particularly valuable in settings where literacy and infrastructure pose challenges, while also supporting better recall and engagement. Despite these benefits, barriers such as low digital literacy, poor connectivity, and data security concerns must be addressed through context-specific and culturally appropriate approaches supported by strong governance and community involvement. Overall, e-consent offers a promising pathway to strengthen equity and ethical standards in research. Future efforts should focus on creating user-friendly, multilingual, and secure platforms, alongside training initiatives and long-term evaluations to guide broader adoption.
